# Editorial: Imaging in the Visual System Disorders

**DOI:** 10.3389/fnins.2022.904854

**Published:** 2022-05-30

**Authors:** Dehui Xiang, Haoyu Chen, Jian Zheng, Vishal Jhanji

**Affiliations:** ^1^School of Electronics and Information Engineering, Soochow University, Suzhou, China; ^2^Joint Shantou International Eye Center, Shantou University and the Chinese University of Hong Kong, Shantou, China; ^3^Department of Medical Imaging, Suzhou Institute of Biomedical Engineering and Technology, Chinese Academy of Sciences, Suzhou, China; ^4^Department of Ophthalmology, University of Pittsburgh, Pittsburgh, PA, United States

**Keywords:** visual system, medical imaging, deep learning, eye disease, ophthalmology

More than 80% of the information we need to perceive the world is delivered by vision. According to the World Health Organization World Report on Vision 2019, there are at least 2.2 billion visually impaired people worldwide, with at least 1 billion possessing a vision impairment that should have been prevented or that is yet to be tackled (World Health Organization, 2019). Visual disorders place significant economic burdens on the patients, their families, and the healthcare system. The diseases of the eye, optic nerve, and brain may cause vision impairment. Early detection and diagnosis of these pathologies would enable clinicians to forestall visual loss.

Imaging is an important technique for diagnosing these diseases (Chen et al., 2019). In the past decades, advances in imaging techniques have provided us tools to improve our understanding of visual system disorders, and facilitate the diagnosis and management of patients with visual system disorders. However, there are still considerable challenges in the field of imaging in visual system disorders, such as precise segmentation of lesions on images, lack of sizeable labeled datasets, correlation of novel imaging biomarkers with clinical diagnosis or management, and correlation of ocular imaging biomarkers with central nervous system disorders or parameters.

An interdisciplinary Research Topic was hosted by the Perception Science section of Frontiers in Neuroscience to cover recent advances in various imaging techniques in diagnosis and management of visual system disorders. In this issue, we aim to address some of these issues surrounding different imaging modalities in visual disorders.

Classification and segmentation of structures and lesions remain challenging for ocular imaging. Recently, artificial intelligence (AI) has dramatically advanced various medical research fields, including imaging segmentation and classification. Numerous deep learning-based ophthalmological image processing algorithms have been proposed to analyze optical coherence tomography (OCT) images, optical coherence tomography angiography (OCTA) images, fundus photographs, and fluorescein staining slit-lamp images. Wang, L. et al. proposed a dynamic multi-hierarchical weighting segmentation network (DW-Net) for the simultaneous segmentation of retinal layers and choroid neovascularization in retinal OCT images. The proposed network was composed of a residual aggregation encoder path to select informative features, a multi-hierarchical weighting connection for the fusion of detailed information and abstract information, and a dynamic decoder path. Their method achieved DICE scores of 0.9484 and 0.9538 for the segmentation of choroidal neovascularization and seven retinal layers, respectively. Wan et al. proposed an optimized Unet to segment parapapillary atrophy in color fundus photos. An edge attention module and a reverse attention module were fused into the UNet (Ronneberger et al., 2015), such that their method achieved a high area under curve (AUC) of 0.9235 over the original UNet of 0.7917. The proposed method worked better for large parapapillary atrophy than small parapapillary atrophy. Xu et al. implemented and investigated the application of a deep learning system based on EfficientNet-B6 in diagnosing pterygium using anterior segment photographs. Their system can judge the presence of pterygium and classify the severity of pterygium. The accuracy of the system was 0.9468.

In the AI-based medical image processing field, it is expensive and time-consuming to obtain the labels of medical data. Therefore, it is advisable to utilize unlabeled data to improve the performance of the algorithms. Meng et al. proposed a multi-scale information fusion network (MF-Net) to segment choroidal neovascularization in retinal OCT images. Their method was evaluated with 1,522 labeled OCT images with choroidal neovascularization and the DICE score was 0.9290. Furthermore, they used a semi-supervised MF-Net with pseudo labels for unlabeled data which improved the DICE score to 0.9307. Wang, T. et al. proposed a semi-supervised multi-scale self-transformer generative adversarial network (Semi-MsST-GAN), which can improve corneal ulcer segmentation in fluorescein staining slit-lamp images by leveraging unlabeled images. Their method achieved a DICE of 0.9093 in evaluating the public SUSTech-SYSU dataset.

This special issue also highlighted a few novel parameters in ocular imaging. Dong et al. used a deep learning-based algorithm for the segmentation of corneal and epithelial thickness on anterior segment-OCT with 1,430 images and characterized the epithelial and corneal thickness changes at different stages of the keratoconus progression. They identified that both epithelial and corneal thickness decreased with the progression of keratoconus, except the epithelial thickness in the scarring stage, which had irregular fluctuation. Ye et al. found that on OCTA, radial peripapillary capillary density, but not peripapillary retinal nerve fiber layer thickness, decreased in pathological myopia compared to simple myopia. Peripapillary capillary density showed the highest AUC for pathological myopia (AUC = 0.962). Furthermore, the best-corrected visual acuity was affected by peripapillary capillary density, axial length, and their interaction.

The eye is a window for the brain and vascular system. Many systemic diseases such as diabetes, cerebral vascular diseases, cardiovascular diseases, and Parkinson's disease may be investigated from ocular parameters (Abràmoff et al., 2010). Zhang et al. used retinal photography and cerebral magnetic resonance imaging (MRI) to evaluate the correlation between retinal microvascular abnormalities in patients with type 2 diabetes and the presence of cerebral small vessel lesions. They found that the degree of diabetic retinopathy and retinal arteriole and venule calibers were associated with MRI burden of cerebral small vessel disease. Zhao et al. used a context encoder network to segment outer retinal layers on OCT images and found that subjects with white matter hyperintensities had a thinner Henle fiber layer, outer nuclear layers, and photoreceptor outer segments, while Parkinson's disease patients had a thicker interdigitation zone and retinal pigment epithelium. Zhou et al. used OCTA and visual evoked potential to study the retinal changes in Parkinson's disease. They found that macular vessel density, but not ganglion cell-inner plexiform layer thickness or the retinal nerve fiber layer decreased in Parkinson's disease. Macular vessel density was also correlated with visual evoked potential. They suggested that retinal microvasculature change may be a biomarker for early diagnosis of Parkinson's disease.

With the development of imaging engineering techniques and the collaboration between clinicians and engineering scientists, we would be able to develop more automatic and precise techniques, which can help clinicians to diagnose and manage patients with visual system disorders, and benefit the patients.

## Author Contributions

DX conceived the idea. HC, JZ, and VJ critically reviewed the manuscript and revised the paper. All authors contributed to the article and approved the submitted version.

## Funding

This work was supported in part by the National Nature Science Foundation of China (61971298, 61771326, and 81871352) by the Suzhou Science and Technology Bureau under Grant SJC2021023, and by the National Key R&D Program of China (2018YFA0701700).

## Conflict of Interest

The authors declare that the research was conducted in the absence of any commercial or financial relationships that could be construed as a potential conflict of interest.

## Publisher's Note

All claims expressed in this article are solely those of the authors and do not necessarily represent those of their affiliated organizations, or those of the publisher, the editors and the reviewers. Any product that may be evaluated in this article, or claim that may be made by its manufacturer, is not guaranteed or endorsed by the publisher.
